# Neighborhood cohesion’s role in supporting perceived mastery and quality of life in older adults

**DOI:** 10.1093/geroni/igaf122.2462

**Published:** 2025-12-31

**Authors:** Inhye Jung, Hyo Jung Lee

**Affiliations:** Yonsei University, Seoul, Republic of Korea; Yonsei University, Seoul, Republic of Korea

## Abstract

Neighborhood cohesion has been linked to the health of older adults by influencing psychological and behavioral well-being. This study examines the role of neighborhood cohesion in older adults’ health, with a particular focus on its support for perceived mastery from a relational autonomy perspective. Specifically, it investigates whether perceived mastery and self-rated health mediate the relationship between neighborhood cohesion and life satisfaction in older adults. Using data from the 2018 Health and Retirement Study (HRS) (N = 2,279), we conducted multiple mediation analyses with PROCESS Macro Model 6 to assess the mediating roles of psychological and health-related factors in this relationship. The findings reveal that higher neighborhood social cohesion is significantly associated with greater perceived mastery (b = 0.104, p < .001), which, in turn, is linked to better self-rated health (b = 0.1172, p < .001) and higher life satisfaction (b = 0.3142, p < .001). The primary mediation pathway (Neighborhood Cohesion → Perceived Mastery → Life Satisfaction) shows a significant indirect effect (b = 0.0327, 95% CI [0.0187, 0.0478]). Additionally, a secondary but significant mediation pathway (Neighborhood Cohesion → Perceived Mastery → Self-Rated Health → Life Satisfaction) further contributes to the overall effect (b = 0.0022, 95% CI [0.0011, 0.0037]). The total indirect effect of neighborhood cohesion on life satisfaction is also significant (b = 0.0403, 95% CI [0.0248, 0.0569]). These results highlight the importance of fostering socially cohesive neighborhoods to enhance psychological resilience, health, and overall life satisfaction among older adults.

